# Gut feelings: gastrointestinal signs in French bulldogs undergoing spinal surgery

**DOI:** 10.3389/fvets.2024.1460092

**Published:** 2024-09-26

**Authors:** Michelle du Toit, Luca Motta

**Affiliations:** ^1^Willows Veterinary Centre and Referral Service, Part of Linnaeus Veterinary Limited, Solihull, United Kingdom; ^2^Northwest Veterinary Specialists, Part of Linnaeus Veterinary Limited, Runcorn, United Kingdom

**Keywords:** French bulldog, gastrointestinal complications, intervertebral disc herniation, hemilaminectomy, ventral slot

## Abstract

**Introduction:**

The French bulldog (FBD) is a brachycephalic breed prone to several neurological conditions, of which intervertebral disc herniation (IVDH) is considerably prevalent. Gastrointestinal (GI) disease is a reported complication in dogs surgically treated for IVDH. The objective of this study was to describe GI signs and their outcome in FBDs surgically treated for IVDH.

**Materials and methods:**

Data regarding the GI signs (vomiting, diarrhoea and regurgitation), their frequency and short-term outcome in FBDs surgically treated for IVDH (cervical, thoracolumbar or lumbar) between January 2017 and April 2023 were obtained from medical records at one institution. Categorical variables were compared using Fisher exact tests, and ordinal/continuous data between categorical groups using Kruskal-Wallis or Mann-Whitney tests.

**Results:**

Ninety-seven FBDs were included for analysis. GI signs occurred in 74/97 (76.3%) FBDs while hospitalised, with 33.8% and 66.2% developing GI signs pre- and post-operatively, respectively. FBDs that developed GI signs had a mean of 4.9 episodes. Diarrhoea was the most common GI sign encountered (51/74) compared to regurgitation (38/74) and vomiting (22/74). Resolution of GI signs occurred within a mean of 2.2 days. Mean duration of hospitalisation post-surgery was 4.6 days in FBDs that developed GI signs versus 3.7 days in FBDs that did not (*p* = 0.033). Anaesthesia length was associated with developing GI signs (p=0.037). Neurological severity, neuroanatomical localisation and surgical procedure were not associated with development of GI signs (*p* = 0.42, *p* = 0.794 and *p* = 1, respectively).

**Conclusion:**

GI signs were commonly encountered in FBDs surgically treated for IVDH and associated with length of anaesthesia and prolonged hospitalisation.

## 1 Introduction

The French Bulldog (FBD) is a brachycephalic breed that has gained increased popularity in the UK over the past 15 years. Second only to the Labrador Retriever in the UK’s most popular breeds in 2022, the Kennel Club recorded 42,538 new registrations in 2022, showing a 6-fold increase since 2013 (1).

FBDs are prone to several neurological conditions with approximately three times higher odds of experiencing spinal cord disorders compared to their non-FBD counterparts (2, 3). Among all FBDs presented for neurological signs at one institution, intervertebral disc herniation (IVDH) accounted for nearly half (45.5%) of the definitive diagnoses made, indicating that IVDH is the most prevalent neurological condition affecting the breed (4). This is higher than in the general dog population with IVDH representing 21% of all neurological conditions (5).

Gastrointestinal (GI) disease is a reported complication in dogs undergoing surgical intervention for IVDH (6–12). Notably, Mehra et al. (8) reported a substantial 47% incidence of GI complications in dogs treated surgically for thoracolumbar IVDH, with diarrhoea emerging as the predominant clinical manifestation (inclusive of vomiting, diarrhoea, regurgitation, melaena and haematochezia). Paran et al. (13) have also highlighted the susceptibility of dogs undergoing general anaesthesia (GA) and magnetic resonance imaging (MRI) for thoracolumbar pathology to gastroesophageal reflux, predisposing them to regurgitation.

Similarly in the human context, GI issues pose a considerable concern for people experiencing acute spinal cord injury (14–16). Notably, these complications account for approximately 11% of rehospitalizations following discharge after acute SCI, highlighting the need for comprehensive management strategies (17–20).

Our retrospective study had two primary objectives. Firstly, to describe the GI signs and their prevalence in FBDs surgically treated for intervertebral disc disease. The second objective was to assess the short-term outcome of these GI signs, specifically regarding their impact on the duration of hospitalisation following spinal surgery and survival to discharge.

## 2 Materials and methods

### 2.1 Case selection

Medical records from one referral hospital were searched for all FBDs that underwent spinal surgery (ventral slot, hemilaminectomy or mini-hemilaminectomy) for the treatment of IVDH from 1 January 2017 to 30 April 2023. Search words included “ventral slot,” “hemilaminectomy,” “mini-hemilaminectomy” and various abbreviations and iterations of the spelling of such words. To be eligible for inclusion, FBDs had to have had an IVDH (protrusion or extrusion) affecting any segment of the spinal column identified via MRI and had to have been treated surgically at the same institution. Included dogs also had to have complete medical records for the duration of hospitalisation. If a case presented multiple times for surgical treatment of IVDH, information from the first presentation only was included in this study.

Dogs with incomplete medical records or with pre-existing conditions that might predispose them to GI signs were excluded. Examples of such pre-existing conditions included hepatic disease, renal disease or previously diagnosed primary GI disease (e.g., inflammatory bowel disease). Dogs with a history of pancreatitis prior to the onset of neurological signs were excluded. Dogs that underwent more than three general anaesthetics within 72 h of the neurosurgical procedure were excluded. Dogs that were euthanised either intra-operatively or within 24 h of surgery were not included in the final cohort for analysis regarding GI signs. Cases in which all pertinent information could not be collected were excluded.

### 2.2 Clinical information

Age, sex, weight, and body condition score (9-point scale) were extracted from the clinical records. The neurological examination was performed by either an ECVN or ECVS diplomate or an ECVN resident in training. Neurological status was assigned using the modified Frankel scale (MFS) (21, 22); grade 0—clinically normal, grade 1—spinal hyperaesthesia only, grade 2—ambulatory para/tetraparesis, grade 3—non-ambulatory para/tetraparesis, grade 4—para/tetraplegic with intact nociception, and grade 5—paraplegic with absent nociception in the pelvic limbs and tail.

### 2.3 Gastrointestinal signs

GI signs were defined as diarrhoea, vomiting and/or regurgitation. Diarrhoea was determined to be present if it was documented as diarrhoea in the clinical notes/in patient charts and/or if there was documentation of a faecal grading score of 5–7/7 using the Purina Faecal Score Chart (23). The presence of GI signs (yes/no) that occurred seven days prior to presentation, as reported by the owner, noted in the referring veterinarian’s records or patient’s history, were abstracted. The length of time of GI signs prior to presentation and current medical therapy for this were recorded. In addition, the administration of a NSAID or corticosteroid prior to presentation was abstracted. Hospital records (including treatment sheets, daily progress notes and client communication logs) were reviewed for GI sign occurrence (yes/no). For each GI sign (diarrhoea, vomiting and regurgitation) and for GI signs in total, the number of episodes, onset (pre- or post-operative) and duration until resolution (in days) was documented. Pre-operative was determined to be from admission to surgery. Post-operative indicated occurrence following surgery. The overall presence of GI signs from admission to discharge was termed “perioperative” (24). Resolution of GI sign was determined as follows: for vomiting and regurgitation; if there was no mention of it for over 24 h and for the remainder of the hospitalisation period. For diarrhoea; if there was no mention of it for over 24 h and the remainder of hospitalisation, or if there was documentation of faeces grading a score less than 5 on the Purina faecal scoring chat while hospitalised.

Medication administered for treatment of GI signs was documented, including the administration of a proton pump inhibitor (PPI). The administration of a non-steroidal anti-inflammatory drug (NSAID) and/or corticosteroid both at presentation and then post-operatively was documented, and whether they were discontinued secondary to the development of GI signs.

### 2.4 Imaging, general anaesthesia, and surgery

MRI (0.4 T, Hitachi APERTO Grande, Steinhausen, Switzerland) was performed on all dogs while under GA for diagnosis of IVDH. The neuroanatomical localisation of the IVDH as determined by advanced imaging and subsequent surgery, was documented. Spinal surgery (ventral slot, hemilaminectomy or mini-hemilaminectomy) was performed by an ECVN or ECVS diplomate or an ECVN or ECVS resident under the direct supervision of an ECVN or ECVS diplomate. Information regarding length of anaesthesia for both imaging and surgery was obtained. Variables that were evaluated to see if there was any impact on the development of GI signs in the post-operative period included length of time under GA (if imaging and surgery were performed under the same GA) and surgical procedure; to avoid bias in this analysis, we excluded dogs that were exhibiting GI signs prior to presentation or during the pre-operative period. Additionally, we grouped mini-hemilaminectomy and hemilaminectomy procedures together as a single category and compared against the ventral slot procedure.

### 2.5 Length of hospitalisation and short-term outcome

For dogs that did not survive to discharge, the reason and whether death was suspected (yes/no) to be associated with the development of GI signs was documented. Length of hospitalisation (in days) was documented as the time from surgery (day 1) to discharge and compared between dogs that had documented GI signs while hospitalised versus those that did not have documented GI signs while hospitalised. It was noted whether the development of GI signs while hospitalised was the direct cause of prolonged hospitalisation.

### 2.6 Statistical analysis

Categorical data were summarised by frequency and percentage and ordinal/continuous data by mean, median and range. Categorical variables were compared to each other using Fisher exact tests, and ordinal/continuous data between categorical groups using Kruskal–Wallis or Mann–Whitney tests adjusted for ties. Significance was taken as *p* < 0.05. Analysis was undertaken using Minitab21 (Minitab LLC, PA, United States 2023).

## 3 Results

### 3.1 Included cases

**Figure 1:**
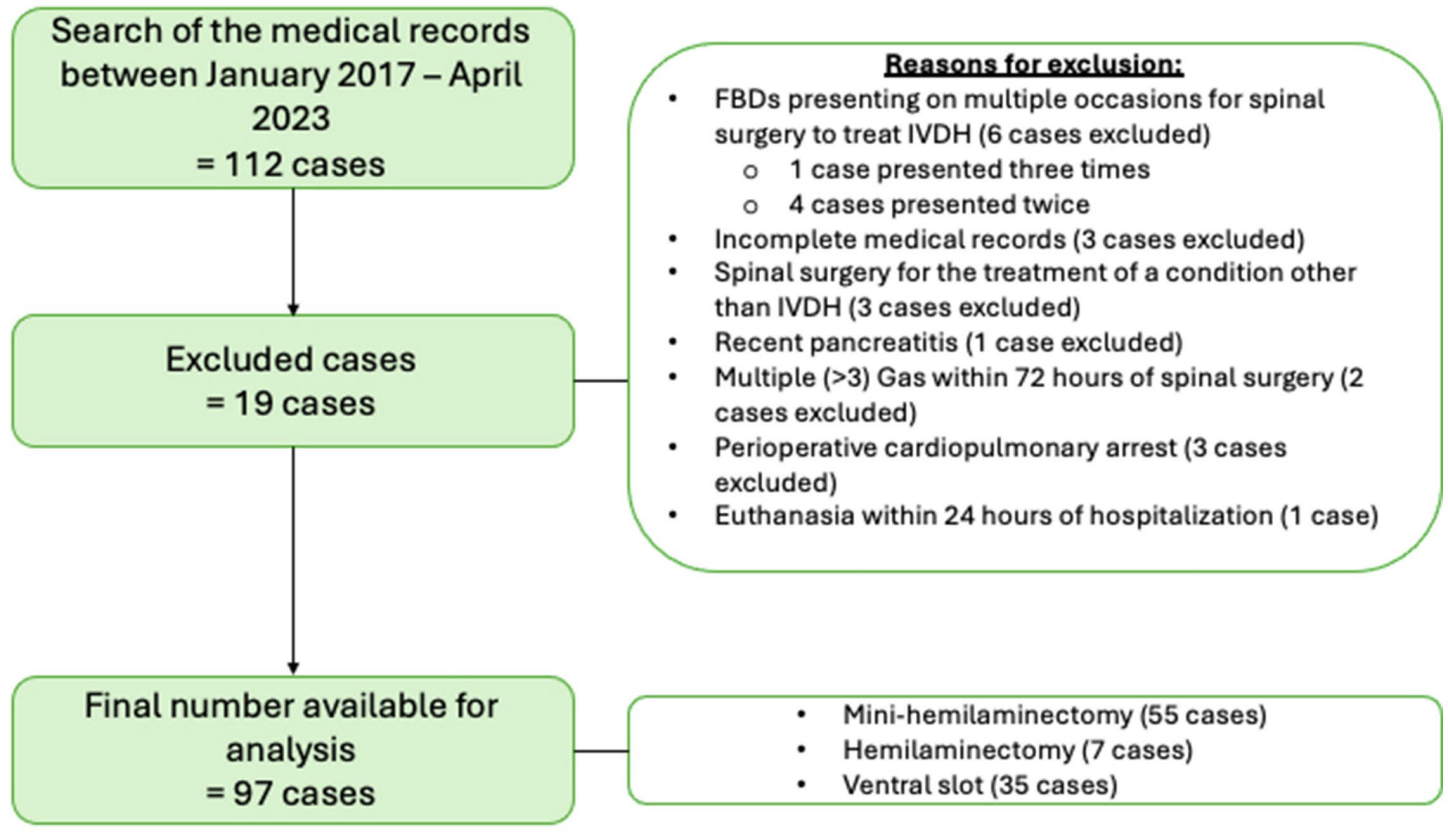
Illustration of excluded cases and reasons resulting in a final case sample of 97 French Bulldogs included for analysis.

One hundred and twelve FBDs underwent spinal surgery between January 2017 and April 2023, some presenting multiple times, resulting in 116 cases of spinal surgery. Figure 1 illustrates the cases excluded for analysis and the reasons why as described in the following text. Nineteen dogs were excluded; 3 dogs had incomplete medical records, 3 dogs underwent spinal surgery for a condition other than IVDH (all of which had surgery for the treatment of thoracolumbar subarachnoid diverticulum), 2 had multiple GAs within 72 h of the initial spinal surgery 1 dog had an emergency ovariohysterectomy for the treatment of a suspected pyometra 2 days prior to mini-hemilaminectomy, 1 dog had repeated imaging and subsequent repeat spinal surgery within 72 h following initial spinal surgery (a repeat MRI was taken 48 h post-operatively due to recurrence of significant cervical spinal pain and identified extradural disc material admixed with haematoma at the original surgical site, this was confirmed with a revision surgery 24 h following MRI), 1 dog was excluded due to a recent history of pancreatitis, 3 dogs died due to cardiopulmonary arrest (CPA) while hospitalised and had insufficient medical records to obtain data for analysis (2 dogs suffered CPA intraoperatively and 1 dog entered CPA while in recovery from GA), 1 dog was euthanised within 24 h of surgery due to the development of progressive ascending descending myelomalacia. Five FBDs presented multiple times for surgical treatment of IVDH (one dog underwent spinal surgery on three separate occasions, and four dogs underwent two spinal surgeries each on separate occasions). All five had documented GI signs in the first presentation but only 3/5 had GI signs documented on subsequent presentations. Only the first presentation for each dog was included in analysis. Our resulting final number for analysis was 97 FBDs.

### 3.2 Clinical information

Of the 97 dogs included for analysis, the mean age was 46.2 months (median 47, range 20–84). Data regarding clinical variables including sex, age, neutering status, weight, body condition score, neurological severity at presentation (MFS) and neuroanatomical localisation can be found in Table 1. None of these clinical variables were found to significantly differ between FBDs that developed GI signs and those that did not.

**Table 1:** Clinical information on FBDs that did not develop (*n* = 23) and did develop (*n* = 74) GI signs while hospitalised.

Clinical variable	No GI signs	GI signs	*p*
Gender			0.570
Male	5 (5%)	24 (25%)	
Male neutered	8 (8%)	28 (29%)	
Female	4 (4%)	10 (10%)	
Female spayed	6 (6%)	12 (12%)	
Weight (kg) (mean [median, range])	13.5 (13.5, 8–21)	13.4 (13, 7.9–19)	0.879
BCS (mean [median, range])	5.4 (5, 4–8)	5.3 (5, 3–8)	0.971
MFS			0.784
1	3 (3%)	12 (12%)	
2	11 (11%)	24 (25%)	
3	5 (5%)	18 (19%)	
4	3 (3%)	13 (13%)	
5	1 (1%)	7 (7%)	
Neuroanatomical localisation†			0.794
C1-C5	8 (8%)	27 (28%)	
C6-T2	0	0	
T3-L3	10 (10%)	25 (26%)	
L4-S1	5 (5%)	22 (23%)	
Surgical procedure			1
Ventral slot	8 (8%)	27 (28%)	
Hemilaminectomy or mini-hemilaminectomy	15 (16%)	47 (48%)	

Percentages are calculated based on the total 97 dogs included for analysis. † confirmed via imaging and surgery.

### 3.3 Gastrointestinal signs

In total, 74/97 (76.3%) FBDs had documented GI signs while hospitalised.

Data regarding the presence of GI signs prior to presentation was available in 96/97 dogs. There were documented GI signs in 18/96 (19%) of those dogs; vomiting in 14 dogs (15%), diarrhoea in 9 (9%) and regurgitation in 1 (1%). Fifteen out of these18 (83%) FBDs continued to have additional GI signs while hospitalised. The mean length of time of GI signs prior to presentation was 2.8 days (median 1 day, range 1–21). Of the 18 dogs that had documented GI signs prior to presentation, only 4 (22%) were receiving medication for this; 1 dog had been administered a single maropitant injection (1 mg/kg, Prevomax, Dechra Veterinary Products), 1 dog was prescribed an anti-diarrhoeal probiotic paste (Prokolin, Protexin Veterinary, Somerset United Kingdom), 1 dog was prescribed butylscopolamine (0.5 mg/kg, Buscopan Compositium solution for injection, Boehringer Ingelheim Animal Health UK Ltd.) and 1 dog had been administered a maropitant injection (1 mg/kg, Prevomax, Dechra Veterinary Products) once off and prescribed ranitidine (brand and dose unavailable).

Of the remaining 78/96 dogs that did not have documented GI signs prior to presentation; 58/78 (74%) had GI signs while hospitalised. Nineteen out of 58 (33%) developed GI signs pre-operatively, while the remaining 39/58 (67%) developed GI signs post-operatively.

Overall, 74/97 dogs developed GI signs while hospitalised. The mean number of documented GI episodes throughout the duration of time hospitalised (vomiting, diarrhoea, and regurgitation) was 4.9 episodes with a median of 3 episodes (range 1–20 episodes).

When looking at the number GI episodes as a whole (diarrhoea, vomiting and regurgitation); 48/75 (65%) of dogs had less than five episodes, 17/74 (23%) had between 5–10 episodes, 7/74 (9.5%) had between 11–20 episodes and 2/74 (3%) had over 20 episodes of GI upset. Diarrhoea was the most common GI sign encountered (51/74; 68.9%) compared to regurgitation (38/74; 51.4%) and vomiting (22/74; 29.7%). Data regarding the occurrence, frequency, onset (pre- or post-operative) and time to resolution of GI signs and their subcategories (diarrhoea, vomiting and regurgitation) in the total number of FBDs that developed GI signs during hospitalisation (*n* = 74) are summarised in Table 2.

**Table 2:** The occurrence, frequency, onset (pre- or post-operative) and duration (in days) of GI signs and their subcategories (diarrhoea, vomiting, regurgitation) in 97 FBDs hospitalised for surgical management of IVDH.

	Any GI signs *n* = 74	Diarrhoea *n* = 51	Vomiting *n* = 22	Regurgitation *n* = 38
Onset of GI sign				
Pre-operatively	25 (33.8%)	20 (39.2%)	7 (31.8%)	15 (39.5%)
Post-operatively	49 (66.2%)	31 (60.8%)	15 (68.2%)	23 (60.5%)
Number of episodes				
1 episode	21	21	10	14
2–4	27	21	10	11
5–9	15	4	1	5
10–19	9	5	1	8
>=20	2	0	0	0
Mean (median, [range])	4.9 (3, [1–20])	3.5 (2, [1–15])	2.6 (2, [1–15])	4.6 (2, [1–15])
Duration GI signs in days†, mean (median, [range])	2.23 (1, [1–12])	2.08 (1, [1–12])	1.65 (1, [1–9])‡	2.34 (1, [1–9])

† *n* = 52 dogs, ‡ *n* = 20, 2 cases did not have data on duration of vomiting episodes. Percentages are calculated from the total number of that subset.

Fifty-six out of 74 (76%) dogs received specific medical therapy for their GI signs during hospitalisation. Medications included maropitant (*n* = 19, 34%), metoclopramide (*n* = 16, 29%), metronidazole (*n* = 1, 2%), omeprazole (*n* = 53, 95%), ondansetron (*n* = 1, 2%), Prokolin probiotic paste (*n* = 22, 39%) and sucralfate (*n* = 2, 4%). Route of administration, brand, dose and duration of therapy varied with each patient.

Fifty-four of the 97 (56%) FBDs received proton pump inhibitors (all omeprazole; 1 mg/kg SID-BID, IV or PO) which were administered either prophylactically (*n* = 19, 35%), therapeutically (*n* = 8, 15%) or both prophylactically and subsequently therapeutically (*n* = 27, 50%). Route of administration, brand, dose and duration of therapy varied with each patient. It was beyond the scope of this study to assess whether medications had any effect of the length of hospitalisation or development of GI signs.

Regarding the administration of an NSAID or corticosteroid prior to presentation; 20/97 had not been administered either whereas, 77/97 dogs had been administered a NSAID or corticosteroid within 24 h prior to presentation. Seventy-two of out 97 (74%) received an NSAID; 61/72 meloxicam, 2/72 carprofen, 1/72 grapiprant, 7/72 robenacoxib and in 1 dog the type of NSAID was not documented. Of those 72 FBDs that had received a NSAID prior to presentation, 54/72 developed GI signs while hospitalised. Thirteen of those 54 dogs had it documented in their clinical records that the NSAID was discontinued as a direct result of the development of GI signs.

Four of the 97 dogs had received a corticosteroid; 2/4 prednisolone (dose range of 0.35–0.45 mg/kg PO sid-bid), 1/4 methylprednisolone (dose 1 mg/kg PO SID) and 1/4 dexamethasone (unknown dose). One out of 97 dogs had received both a NSAID and corticosteroid within 24 h prior to presentation (dexamethasone and meloxicam at unknown doses).

One of the FBDs that had been administered a corticosteroid (prednisolone) prior to presentation was prescribed a NSAID (meloxicam) post-operatively following a wash out period of 48 h, but this was soon discontinued due to the development of GI signs post-operatively. The remaining three dogs administered corticosteroids prior to presentation had these discontinued pre-operatively and no further corticosteroid or NSAID was prescribed. Only one of those three developed GI signs post-operatively.

Seventy-six out of 97 (78%) FBDs were prescribed a NSAID as part of their post-operative analgesia; 1/76 (1%) received carprofen (2 mg/kg PO SID, Rimadyl, Zoetis UK Limited), 1/76 (1%) received grapiprant (2 mg/kg PO BID, Galliprant, Elanco UK AH Limited), 65/76 (86%) received meloxicam (0.1–0.2 mg/kg IV, SQ or PO SID, Metacam, Boehringer Ingelheim Animal Health UK Ltd.) and 9/76 (12%) received robenacoxib (1–2 mg/kg IV or PO SID, Onsior, Elanco UK AH Limited). Of these 76 cases, 56 FBDs (74%) developed GI signs while hospitalised. The NSAID was withdrawn in 17 dogs (22%), with the documented reason being the onset of GI signs during their hospitalisation. If the FBD had been on a NSAID prior to presentation and had been prescribed a NSAID as part of the post-operative analgesia, the same NSAID as used prior to presentation was prescribed.

Two dogs received corticosteroids while hospitalised and they both developed GI signs while hospitalised, albeit only 1 episode each.

### 3.4 Imaging, general anaesthesia, and surgery

The anaesthetic protocol varied between dogs and was determined by the anaesthetist overseeing the case. The neuroanatomical localisation was C1-C5 in 35 dogs, T3-L3 in 35 and L4-S1 in 27 dogs. Fifty five out of 97 dogs (57%) underwent a mini-hemilaminectomy, 7/97 (7%) underwent a hemilaminectomy and 35/97 (36%) a ventral slot.

There was no statistical significance (*p* = 1.000) in the development of GI signs when comparing the type of surgical procedure performed (mini-hemilaminectomy/hemilaminectomy [65%] versus ventral slot [68%]).

There were complete records regarding the total length of time under GA for both imaging and surgery available in 90 dogs. The mean length of total time under GA was 251 min (median 235 min, range 145–420 min).

For the FBDs that (1) had imaging and surgery carried out under the one GA and (2) did not have GI signs pre-operatively (*n* = 31), 10/31 (32%) did not develop GI signs post-operatively and had a mean length of time under GA of 222 min (median 212 min, range 160–315). The 21/31 (68%) that did develop GI signs post-operatively had a mean length of time under GA of 269 min (median 270 min, range 170–420). This was found to be significantly different (*p* = 0.037).

For the FBDs that (1) had two separate anaesthetic events for both imaging and surgery and (2) did not have GI signs pre-operatively (*n* = 28); 10/28 (35.7%) did not develop GI signs post-operatively and had a mean accumulative time under GA of 247 min (median 230 min, range 175–300). Whereas the 18/28 (64.3%) that did develop GI signs post-operatively had a mean accumulative time under GA of 250 min (median 230 min, range 175–375).

There was no significant difference in the development of GI signs in the post-operative period whether FBDs had two separate GAs for imaging and surgery or whether they had just one GA period (*p* = 0.791).

### 3.5 Length of hospitalisation and short-term outcome

Of the original 116 cases, including the excluded cases for analysis, no dog was euthanised or died as a result of any documented GI signs. Of the 97 dogs included for analysis, all dogs survived to discharge.

Of the 74 dogs that developed GI signs while hospitalised, data on resolution of GI signs were available in 52 cases. Resolution of GI signs occurred within a mean of 2.2 days (median 3 days, range 1–20).

The mean duration of hospitalisation from surgery to discharge was 4.4 days (*n* = 97) with a median of 4 days (range 1–17). Those that developed GI signs while hospitalised (*n* = 74) had a mean duration of 4.6 days of hospitalisation following surgery (median 4 days, range 1–17) whereas those that did not develop GI signs (*n* = 23) had a mean duration of 3.7 days (median 3 days, range 2–9). A Mann–Whitney test adjusted for ties found this difference to be significantly different (*p* = 0.033).

Of the 74 dogs that had documented GI signs while hospitalised, we had available follow up data regarding resolution of GI signs in 54/74 dogs (74%). Forty-seven dogs out of 54 (87%) did not have GI signs within 24 h prior to discharge. Seven out of 54 dogs (13%) were still showing GI signs within 24 h of discharge, one of which was re-admitted 3 days following discharge for medical management of ongoing GI signs. In two cases, despite having no GI signs within 24 h of discharge, documented telephone updates with the owners reported GI signs while at home; both cases saw their primary veterinarian regarding ongoing treatment of these signs and no further follow-up was available.

Three dogs had prolonged hospitalisation as documented in their clinical records specifically due to the development of GI signs, despite neurological improvement. One dog experienced regurgitation while hospitalised and was discharged 7 days post-operatively due to neurological improvement, but regurgitation had not resolved. This dog was re-admitted 3 days following discharge due to persistent GI signs (regurgitation) and remained hospitalised for the treatment of this for a further 4 days. The second dog was hospitalised an additional 32 h due to GI signs, this dog also had a suspected urinary tract infection which may have contributed to extended hospitalisation. This dog was free of GI signs for 24 h prior to final discharge. The third dog was hospitalised an additional 24 h due to GI signs (vomiting), although there was a noted improvement in GI signs within those additional 24 h.

## 4 Discussion

The present study was focused on GI signs and their short-term outcome in FBDs undergoing spinal surgery for IVDH. Our findings demonstrate a high prevalence of GI signs in FBDs following spinal surgery, with approximately three-quarters of cases experiencing GI signs while hospitalised. The majority of dogs (66%) in our cohort developed GI signs during the post-operative period and the most common GI sign encountered was diarrhoea (69%) compared to vomiting and regurgitation which occurred in 30 and 51% of FBDs, respectively. These results align with current literature findings which highlight the susceptibility of dogs to GI complications following GA and surgery for intervertebral disc disease such as gastroesophageal reflux, regurgitation and diarrhoea (8, 9, 12, 13, 25).

There are several factors implicated in the development of GI complications following acute spinal cord injury (SCI) in both the veterinary and human literature. The potential dysregulation of the sympathetic-parasympathetic nervous system dynamics, post-injury systemic inflammation, and immune suppression may contribute to GI complications, particularly in patients experiencing more severe SCI (8, 11, 14, 26, 27). Additionally, there is a broader spectrum of influences indirectly related to SCI that potentially predispose to GI issues, such as stress associated with hospitalisation, surgery, and GA (12, 28–30). Furthermore, the perioperative use of opioids and ulcerogenic medications such as corticosteroids and NSAIDs may significantly contribute to GI vulnerability (12, 25, 31–34).

There are very few studies specifically addressing GI complications in dogs undergoing surgical treatment for IVDH. Mehra et al. (3, 8), reveal a GI complication rate of 46–47%, surpassing extrapolated rates from previous studies of 15–39% (7, 10, 11, 32, 35). However, our current investigation presents a notably higher complication rate of 76%. We believe this may be attributed to our exclusive focus on FBDs. It is plausible that FBDs are inherently predisposed to GI complications due to their brachycephalic conformation as evidenced by Poncet et al. (36) and Kaye et al., (37). These studies also highlighted a higher prevalence of GI tract disorders in FBDs compared to other brachycephalic breeds such as the English Bulldog and Pug. However, unlike our cohort which focused on FBDs undergoing spinal surgery, these prior studies centred on dogs presented for surgical treatment of respiratory issues associated with brachycephalic obstructive airway syndrome (BOAS). It is conceivable that the high incidence of BOAS in FBDs in general would render the dogs in our study population inherently more susceptible to GI complications than the general population of non-brachycephalic dogs presenting for surgical treatment of IVDH. We did not assess the presence or severity of BOAS in the FBDs undergoing spinal surgery in our population; however, given that almost 1 in 5 FBDs experience respiratory compromise when presented for IVDH (38) or other unrelated problems (39), future studies regarding FBDs would benefit from incorporating BOAS assessment into their study design and analysis.

FBDs in the present study that developed GI signs were hospitalised longer in the post-operative period than dogs that did not. However, when analysing clinical notes and daily hospital records, there were only three cases where it was documented that hospitalisation was purposefully extended as a direct result of the development of GI signs. In one of the cases, the FBD was initially discharged due to improving neurological status despite the persistence of GI signs (regurgitation) however, it was re-admitted 3 days following discharge for medical management of ongoing GI signs and hospitalised for a further 4 days. The second case had hospitalisation prolonged a further 32 h for medical management of ongoing vomiting and regurgitation. This patient also had a suspected urinary tract infection that may have contributed to delayed discharge. The third dog was hospitalised an additional 24 h due to ongoing vomiting, which subsequently improved during the additional day in hospital. There was no other case for which GI signs were the documented reason for prolonged hospitalisation. However, although it may not be documented in the clinical notes, we cannot say with certainty that GI signs were not a factor considered or discussed with owners when determining a time for discharge for other cases.

Despite the high prevalence of GI signs observed in our study cohort, signs were reasonably mild with 64.9% of cases experiencing less than 5 episodes of any GI disturbance while hospitalised. We also found a favourable short-term outcome, with all 97 dogs surviving to discharge and most experiencing a resolution of GI signs within a relatively short duration (mean of 2.2 days). Our primary focus was to characterise the clinical manifestation of GI signs in this cohort, necessitating the exclusion of patients lacking appropriate medical records during the perioperative period to ensure data accuracy. Consequently, cases that were euthanised or died during or shortly after surgery were omitted from our analysis. It is worth noting, however, that when reviewing the cases prior to exclusion, these dogs were euthanised or passed away due to causes unrelated to GI signs (two due to CPA in the peri-anaesthetic period and one due to the development of progressive myelomalacia). Mehra et al., (8) found a GI mortality rate following thoracolumbar spinal surgery of <1%, and a relevant VetCompass study (40) determined that GI signs accounted for only 1% of GA/sedative-related death among 157,318 dogs undergoing GA or sedation. Therefore, given that all our 97 FBDs were successfully discharged despite a high incidence of GI signs while hospitalised, and in combination with the available literature, we believe it is reasonable to conclude that severe and life-threatening GI complications in dogs surgically treated for IVDH during the perioperative period is possible yet exceedingly rare, even in a brachycephalic breed predisposed to GI disturbances.

For dogs undergoing a single anaesthetic event for imaging and surgery, those with GI signs had significantly longer anaesthetic events than those without GI signs. This was not surprising as there are multiple studies providing evidence that longer duration of GA is associated with the development of GI signs such as gastroesophageal reflux, regurgitation, vomiting, post-operative diarrhoea and functional ileus (8, 29, 40, 41). However, the anaesthetic protocols varied for each dog, and it was beyond the aims of our study to explore GA related variables such as hypotension and opioid use, and their influence on the development of perioperative GI signs. To limit confounding factors, we excluded dogs that had multiple GAs (>3 GAs) within 72 h of the surgical procedure.

The medical management of GI signs while hospitalised varied between patients and was not standardized. Given the retrospective nature of the study and the lack of standardisation regarding medical treatment of GI signs while hospitalised, it was not possible to obtain adequate data on the effectiveness of varying approaches to medical management. Many cases received PPIs, some of which were prescribed prophylactically as part of the premedication or as part of their post-operative/post-anaesthetic medication protocol. While the prophylactic administration of gastroprotectants has been common practice in the past few decades (42, 43), there is little evidence to suggest that it is beneficial in reducing GI complications following SCI (25, 44). In fact, emerging evidence suggests adverse associations with prophylactic use of gastroprotectants and heightened GI complication rates during hospital stays (30, 42, 44).

In the past, corticosteroids were frequently employed in the initial management of SCI. However, numerous studies have highlighted the associated risk of considerable GI adverse effects (31, 32, 45), leading to a decline in its favour in the treatment of SCI. Instead, NSAIDs have become more commonly utilised, although they too are linked to adverse GI effects (12, 33). This shift is evident in our study, where the majority of FBDs received NSAIDs as part of their post-operative analgesia, while only two were prescribed short courses of corticosteroids (Prednisolone 0.4 mg/kg PO SID). The limited number of corticosteroid-treated dogs compared to those administered NSAIDs precluded reasonable statistical analysis.

Our study is subject to several limitations. Primarily, its retrospective nature resulted in inconsistent documentation of GI signs throughout, potentially resulting in a less accurate depiction of the characteristics and severity of GI signs developed while hospitalised. While we were able to obtain sufficient follow-up data regarding the resolution of GI signs in 54/74 dogs, the retrospective nature of our analysis hindered our ability to gather specific post-discharge follow-up information regarding GI signs in several dogs. In addition, there is variability in treatment approaches (including those related to IVDH, perioperative analgesia and GI symptom prevention or treatment) among dogs. This lack of standardization is another significant limitation to our study. Given that FBDs in general are commonly affected with clinical signs of GI disease (2, 3, 36, 46), we excluded dogs with previously diagnosed conditions that would have further contributed to a predisposition to GI upset while hospitalised, such as IBD, chronic or recent pancreatitis, endocrinopathies and hepatopathy. However, a limitation of this study is the possibility that some FBDs included for analysis had undiagnosed concurrent GI disease which may have increased the prevalence of GI signs in this cohort.

In conclusion, FBDs surgically treated for IVDH (cervical, thoracolumbar or lumbar) exhibit an increased incidence of GI signs during hospitalisation, particularly in the post-operative period. Additionally, and unsurprisingly, FBDs that developed GI signs in the post-operative period tended to be hospitalised longer than those that did not. Despite the increased occurrence, instances of mortality attributed to GI complications remain exceptionally rare. Our results indicate that increased duration of anaesthesia was associated with the development of GI signs, whereas factors such as neurological severity, neuroanatomical localisation, and the specific neurosurgical procedure (ventral slot vs. mini-/hemilaminectomy) show no significant association with GI sign development during hospitalisation. Given the widespread popularity of the FBD breed and their frequent presentation to specialist referral centres, our findings serve as valuable information for informing owners about potential complications and challenges following spinal surgery.

## Data Availability

The raw data supporting the conclusions of this article will be made available by the authors upon request, without undue reservation.
